# Evaluation of Mosquito Blood Meals as a Tool for Wildlife Pathogen Surveillance

**DOI:** 10.3390/pathogens14080792

**Published:** 2025-08-08

**Authors:** Samantha M. Wisely, Carson W. Torhorst, Sebastian Botero-Cañola, Hannah Atsma, Nathan D. Burkett-Cadena, Lawrence E. Reeves

**Affiliations:** 1 Department of Wildlife Ecology and Conservation, University of Florida, Gainesville, FL 32611, USA; carsontorhorst1@gmail.com (C.W.T.); sebastian.botero@ufl.edu (S.B.-C.); 2 Florida Medical Entomology Laboratory, University of Florida, Vero Beach, FL 32962, USA; hannah.atsma@gmail.com (H.A.); nburkettcadena@ufl.edu (N.D.B.-C.); lereeves@ufl.edu (L.E.R.)

**Keywords:** environmental DNA, eDNA, insect DNA, iDNA, wildlife epidemiology, xenomonitoring, xenosurveillance

## Abstract

Mosquito blood meals provide a biological sample of host blood which can then be used in downstream applications including host–pathogen detection. We conducted DNA barcoding to identify the host species of blood meals from 4557 blood engorged mosquitoes collected in south central Florida, USA. We identified 314 blood meals from invasive wild pigs, 219 wild turkey blood meals, and 1046 white-tailed deer blood meals. From a subset of these host blood meals, we used molecular assays to detect the nucleic acids of Torque teno sus virus 1 (TTSuV1) in wild pig blood meals, Lymphoproliferative virus (LPDV) in wild turkey blood meals, and bluetongue virus (BTV) in white-tailed deer blood meals. None of these wildlife pathogens are transmitted by mosquitoes, but viral nucleic acids circulate in the peripheral blood of host species during or post infection. Prevalence of TTSuV1 in wild pig blood meals was 34%; in wild turkey blood meals the prevalence of LPDV was 2.7%, and BTV prevalence in blood meals of white-tailed deer was 3.6%. These prevalence values were similar to estimates obtained from peripheral blood collected directly from these hosts in Florida. Our analysis suggests that mosquito blood meals are a valuable sampling tool for the detection of wildlife pathogens. We suggest that this type of exogenous xenosurveillance, using mosquitoes to infer information about the vertebrate host, is distinct from endogenous xenosurveillance in which the goal is to understand the role of the arthropod in vectoring a pathogen.

## 1. Introduction

Noninvasive methods for detecting pathogens of wildlife have proliferated in the last 25 years [1]. Advances in biotechnology have increased assay sensitivity, allowing for nucleic acids or proteins of pathogens to be detected at very small quantities from a variety of environmental substrates rather than from restrained animals. For example, assays have been developed for pathogen detection in feces for a variety of disease systems including prion proteins in cervids [2], viral RNA in rodents [3], and bacterial DNA from carnivores [4]. Environmental sampling of pathogens has been developed for other pathogen shedding routes including saliva, urine, feathers, hair, and skin [1]. Xenosurveillance, or the use of blood meals from blood-feeding invertebrates, has also been successfully used to detect pathogens, even when those pathogens are not vector-borne, but directly transmitted from vertebrate to vertebrate [5]. We specifically define this use of arthropod vectors to detect pathogens circulating in the peripheral blood of vertebrates as exogenous xenosurveillance, and distinguish it from endogenous xenosurveillance which we define as detecting vector-borne pathogens in the midgut or salivary glands of arthropod vectors.

Hematophagous invertebrates act as living syringes as they take blood meals from their vertebrate hosts. These blood samples can then be used in downstream analyses. Leeches, tsetse flies, blowflies and mosquitoes have previously been used to detect nucleic acids of wildlife pathogens [6] or host antibodies to pathogens [7,8] that circulate in the vertebrate host’s peripheral blood system. Importantly, these pathogens are not vector-borne; they are simply present in the abdomen of the blood-feeding invertebrate as part of the host blood meal [9]. An important consideration for this type of sampling, however, is that the pathogen or biomarker of the pathogen circulates in the host’s peripheral blood at the time of blood feeding. Ultimately, the benefit of this type of vertebrate blood sampling is that the vertebrates do not need to be captured and handled which reduces expenses and risks of injury or death to both wildlife and the humans handling them. In addition, a large number of peripheral blood samples from vertebrates can be gathered in a relatively short amount of time [9]. Despite the potential usefulness of xenosurveillance, this non-invasive method of collecting a peripheral blood sample has not been validated for many pathogens or in many ecosystems.

To evaluate the use of mosquitoes for xenosurveillance in the subtropical ecoregion of south central Florida, we focused on three disease systems with properties that make them useful for the evaluation of exogenous xenosurveillance. Each of these pathogens is endemic to Florida and their vertebrate hosts are regionally widespread and abundant. In addition, each of these viruses circulates in peripheral blood systems of their hosts at some point in the progression of the infection [10,11,12] making the viral nucleic acid available for uptake during blood feeding by mosquitoes.

Torque teno sus virus 1 (TTSuV1) is a small, circular DNA virus in the family Anelloviridae. The virus has a global distribution in wild and domestic pigs (*Sus scrofa*), and is ubiquitous within populations including invasive wild pigs in North America [13,14]. It is directly transmitted both through vertical and horizontal routes [15], but it is not thought to be transmitted by arthropod vectors. Lymphoproliferative virus (LPDV) is a retrovirus (Family Retroviridae) that infects poultry, especially domestic and wild turkeys (*Meleagris gallopavo*). In some infected wild turkeys, LPDV can cause lymphoid neoplasia resulting in morbidity and mortality, but many animals remain asymptomatic. The prevalence of this virus is highly variable among populations in the southeastern USA [16]. While direct transmission routes in domestic turkeys have been demonstrated, other routes of transmission have not been studied [17], yet mosquitoes are not thought to vector the pathogen. Bluetongue virus (BTV) is an RNA virus in the Reoviridae family that causes hemorrhagic disease in wild and domestic ruminants. This arbovirus has a nearly global distribution and is transmitted by various species of Culicoides biting midges [18]. In the USA it is a major cause of morbidity and mortality in white-tailed deer (*Odocoileus virginianus*) [19]. Each of these viruses and their vertebrate hosts occur in south central Florida where the study took place.

In this study, our aim was to determine if xenosurveillance using mosquito blood meals could detect three pathogens of varying prevalence from three different host species. As all viruses were detected in our study, our second aim was to compare the prevalence of the viruses in the mosquito blood meals to the prevalence of the viruses in host populations at or near the sampling location.

## 2. Materials and Methods

### 2.1. Mosquito Collection

**Figure 1:**
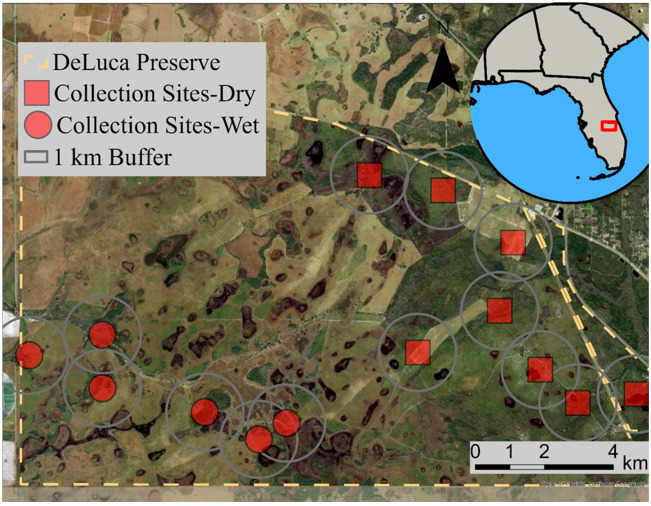
Satellite imagery of the Deluca Preserve, Florida, USA. Red circles and squares indicate sampling locations. We have drawn 1 km radius buffers around each sampling location, equivalent to the home range size of female invasive wild pig in the region [24].

Mosquito collection occurred over 50 days at 14 sites that spanned both the dry and rainy season from January to October 2022 at the University of Florida DeLuca Preserve (Figure 1) [20]. This 11,000 ha property was, in part, a working cattle ranch in the subtropical climate of Osceola County in south-central Florida and part of the pine flatwood ecosystem [21]. Blood-fed mosquitoes were collected from four habitat types (Florida scrub, upland pine flatwoods, marsh, and citrus grove) using battery powered aspirators and pop-up resting shelters [20]. These methods of mosquito collection have previously been shown to target blood-fed mosquitoes which typically are not attracted to conventional mosquito lures like CO_2_ or light which are used to trap blood-seeking mosquitoes [22,23].

The average distance between sampling locations was 1.6 km and the closest locations were 0.8 km apart. During the dry season samples were collected from eight locations during nine sampling events that lasted five days each. During each day of sampling, aspirators were operated for 10 min at every site, and mosquitoes were collected from the two to three resting shelters installed at each site. During the rainy season, six locations were sampled during one event that lasted five days and consisted of daily aspirator sampling at each location. Following collection, blood-fed mosquitoes were killed by placing them in a cooler with dry ice and then frozen at −20 °C. Mosquitoes were identified to species using a dissecting microscope and taxonomic keys [20,25].

### 2.2. Nucleic Acid Extraction from and Identification of Mosquito Blood Meals

Blood from the abdomens of fully engorged, blood-fed mosquitoes was transferred to Flinders Technology Associates (FTA) cards (QIAcard FTA Classic, Qiagen, Hilden, Germany) by extruding the abdominal contents using a sterile pipette tip onto the FTA card [26]. The cards were stored at room temperature until DNA extraction. For identification of blood meal species, DNA was extracted from two 1 mm circular punches of each blood meal using the HotSHOT method of preparation [27]. The species from which each blood meal was derived was identified using DNA barcoding methods previously described [20,28]. This method of blood collection and storage has previously been shown to have sufficient quantity and quality for multiple nucleic acid extractions from a single blood meal [9,26].

From each set of blood meals from wild pigs and wild turkeys, 110 blood meals were randomly selected for pathogen detection of DNA viruses. DNA was reextracted from new FTA card punches (two 1 mm punches) of the same blood meals using the Qiagen Puregene Blood and Tissue Kit (Qiagen, Hilden, Germany) and each sample was incubated at room temperature for three days in 600 µL of PureGene Cell Lysis Solution to lyse cells bound to the FTA card. In addition, 20 µL of Proteinase K (MilliporeSigma Life Sciences Company, Burlington, MA, USA) were added to each sample and incubated at 56 °C for 24 h to digest the proteins released during cell lysis. Following protein digestion, total DNA was extracted according to the manufacturer’s protocol. DNA from wild pig and wild turkey blood meals was resuspended in 50 µL and 100 µL, respectively, of PureGene DNA Hydration Solution (Qiagen, Hilden, Germany). DNA was lightly vortexed in PureGene DNA Hydration Solution and stored at room temperature for 24 h to allow the pelleted DNA to fully elute into solution and then stored at 4 °C until quantified.

From a subset of the mosquito blood meals identified as white-tailed deer (*n* = 165), viral RNA was extracted from the FTA cards using the Qiagen QIAamp Viral RNA Mini Kit (Qiagen, Hilden, Germany). To extract RNA, we followed the modifications to the manufacturer’s recommended protocol [29]. The modification included placing each sample in 150 µL of RNase Free PBS and 600 µL of Buffer AVL and incubating at room temperature for a minimum of 10 min prior to extraction. The rest of the RNA extraction protocol followed the manufacturer’s recommendation. Following extraction, RNA was eluted in 50 µL of warmed (56 °C) Buffer AVE (Qiagen, Hilden, Germany) and stored at 4 °C until quantified.

DNA and RNA were quantified using a NanoDrop 2000 spectrophotometer (Thermo Fisher Scientific, Waltham, MA, USA). Following quantification, DNA and RNA were stored at 4 °C until PCR amplification. Following amplification DNA was placed in long term storage at −20 °C and RNA at −80 °C.

### 2.3. Molecular-Based Pathogen Detection from Mosquito Blood Meals

Full details of reaction conditions and primer sequences are provided in the Supplementary Materials for each of the three pathogen detection assays. To detect TTSuV1 in mosquito blood meals derived from wild pig, a conventional PCR was used to detect a 309-base pair (bp) product of the untranslated region (UTR) of the TTSuV1 genome [30]. For each assay, positive and negative controls were used to verify reaction function; negative controls were molecular grade water in place of DNA template. For the TTSuV1 assay, positive controls contained viral DNA of TTSuV1 extracted from blood of infected wild pigs sampled in Florida [14]. To detect LPDV in wild turkey blood meals, a conventional PCR detected a 415 bp section of the proviral genome spanning the p31 and CA genes [17,31]. Positive controls of DNA extracted from the blood of LPDV-infected wild turkeys were provided by Southeastern Cooperative of Wildlife Disease Study (SCWDS) at the University of Georgia.

For TTSuV1 and LPDV assays, products of conventional PCRs were visualized on an agarose gel using 1.5% TAE agarose (Genesee Scientific, Morrisville, NC, USA) loaded with RedView DNA Gel Stain (ABP Biosciences, Beltsville, MD, USA). For each sample 1 µL of amplified DNA template and 5 µL of 6× loading dye (Thermo Fisher Scientific, Waltham, MA, USA) were mixed and added to their respective well in the gel and the gel was visualized using the UVP Gel Studio Plus (Analytik Jena, Jena, Germany).

To detect viral RNA of BTV from mosquito blood meals derived from white-tailed deer, we used a reverse transcriptase qPCR (RT-qPCR) with primers and probe described in [32]. We used both an internal positive control (VetMAX Xeno Internal Positive Control—VIC, Thermo Fisher Scientific, Waltham, MA, USA) and an external positive control using isolates of BTV cultured from the spleen of Florida white-tailed deer [33]. A sample was deemed BTV positive if the C_t_ value was ≤38. Any sample whose internal positive control did not amplify underwent a 1:1 (water:template RNA) and 1:2 dilution to reduce sample inhibition and was reamplified using the same reaction procedure.

Apparent pathogen prevalence for xenosurveillance was reported as the number of positive molecular assays for a particular pathogen divided by the total number of blood meal samples tested for the relevant host species. We estimated 95% confidence intervals of prevalence estimates using the Wilson score interval which addresses the uncertainty of the prevalence estimate based on sample size [34].

To assess if xenosurveillance prevalence estimates reflected apparent prevalence estimates from host surveillance, we used a 2 sample z-test to compare the proportion of positive samples collected from xenosurveillance vs. host surveillance (i.e., direct collection of blood or spleen from host animals). Host surveillance data came from previously published studies or from the Florida Fish and Wildlife Conservation Commission. These studies were conducted in the same region and within five years of the xenosurveillance study.

## 3. Results

**Figure 2:**
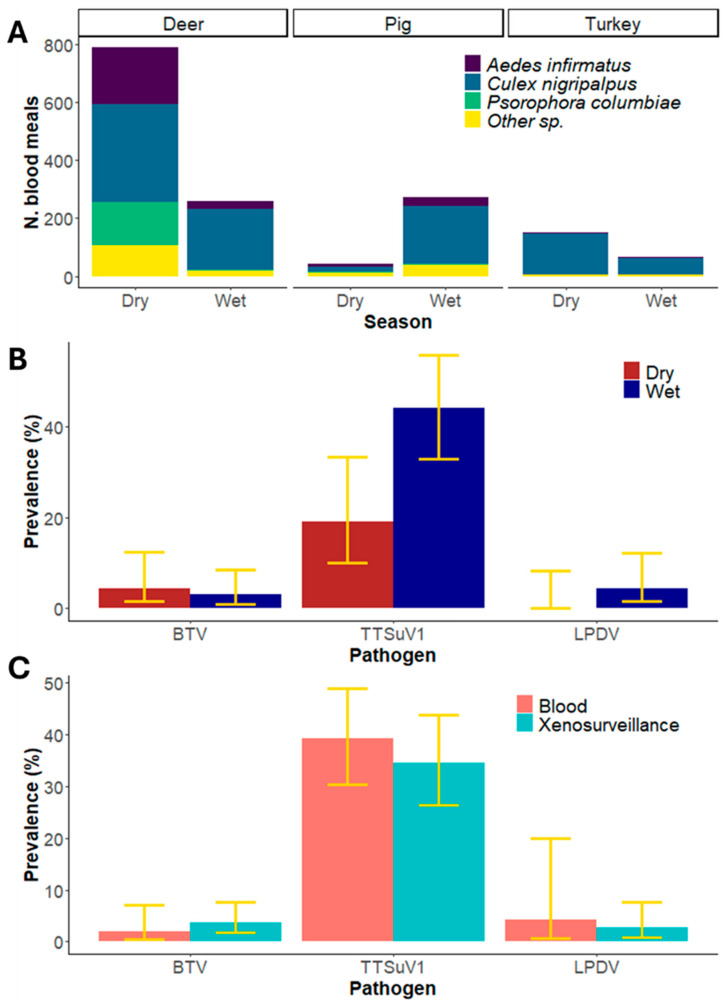
Host and pathogen analysis from mosquito blood meals. (**A**) The total number of collected blood meals for each target host, the contribution of mosquito species to this sample, and sample size for each season (due to differences in sampling effort during each season, these values are not indicative of sampling efficiency). (**B**) Apparent prevalence estimated by xenosurveillance during the dry and wet season for each focal pathogen. Yellow bars indicate 95% confidence intervals. (**C**) Overall apparent prevalence in the region estimated by xenosurveillance compared to estimates from peripheral blood drawn directly from hosts. Yellow bars indicate 95% confidence intervals.

Over the sampling period, we collected 54,637 mosquitoes of which 4557 were blood-fed [20]. Of these, 1581 (35%) blood meals were identified by DNA barcoding as coming from the target host species. A total of 1047 blood meals were obtained for deer, representing both the dry (788; 75%) and wet (259; 25%) seasons. Three mosquito species accounted for the majority of these blood meals (Figure 2): *Culex nigripalpus* (52%), *Aedes infirmatus* (19%) and *Psorophora columbiae* (14%). Wild pigs comprised 314 blood meals, with the majority of these (272; 87%) collected during the wet season despite the lower collection effort during this season. Similar to deer, *C. nigripalpus* (63%) and *A. infirmatus* (13%) provided most of the wild pig blood meals. Wild turkey comprised 220 blood meals, representing the dry (152; 69%) and wet (68; 31%) seasons. For wild turkey, *C. nigripalpus* also provided the majority of blood meals (90%; Figure 2; Supplementary Table S1).

In deer, BTV RNA was detected in six of the 165 blood meals tested, for an overall apparent prevalence of 3.6% (1.7–7.7%). Apparent prevalence in each season, was 4.5% (1.5–12%) and 3% (1–8.6%) for the dry and wet season, respectively (Supplementary Table S2). The prevalence of BTV estimated from peripheral blood collected from white-tailed deer in Florida from 2017 to 2019 was 2.0% (0.5–7.1%; two of 99 samples [35]) and not statistically significantly different from xenosurveillance estimates (z-value = 0.7, *p* = 0.46, Figure 2).

From a sample of 110 wild pig blood meals, 38 had viral DNA from TTSuV1, resulting in an overall apparent prevalence of 34.5% (26.3–43.8%). During the dry season, the apparent prevalence was 19% (10–33%), and 44% during the wet season (33–56%; (Supplementary Table S2). In 2017 and 2018 the prevalence of TTSuV1 estimated from peripheral blood drawn from wild pigs in Highlands County, Florida (~55 km from our study area) was 39.2% (40 positive samples of 102 tested, 30.3–48.9%; [14]). There was no significant difference between both estimates (z-value = 0.7, *p* = 0.48, Figure 2).

In wild turkeys, LPDV RNA was detected in three of the 110 assessed blood meals, resulting in an overall apparent prevalence of 2.7% (0.9–7.7%). The virus was not detected in the samples from the dry season, while it presented an apparent prevalence of 4.4% (1.5–12%) during the wet season (Supplementary Table S2). The prevalence of LPDV estimated from peripheral blood drawn from 23 wild turkeys in the same study area in 2023 was 4.3% (0.7–20%; Florida Fish and Wildlife Conservation Commission, unpublished data), not significantly different from the xenosurveillance apparent prevalence estimate (z-value = 0.4, *p* = 0.68, Figure 2).

## 4. Discussion

Mosquitoes have been shown to be an excellent source of peripheral blood from wild pigs in this ecoregion [9]. In addition to wild pigs, the current study highlights the utility of xenosurveillance for monitoring two additional game species of management concern, wild turkey and white-tailed deer. The sample sizes of each host species acquired from blood fed mosquitoes and the sample quality of both extracted viral RNA and DNA using our xenosurveillance methodology were sufficient to also detect three commonly circulating pathogens endemic to these vertebrate host species: TTSuV1 in invasive wild pigs, LPDV in wild turkeys, and BTV in white-tailed deer. In addition to detecting these viral pathogens, we were able to estimate the prevalence of these viruses in mosquito blood meals and by extension in the vertebrate host populations. In each case, the pathogen prevalence in the blood meal reflected the apparent prevalence of each virus circulating in local populations as measured by directly sampling the hosts, suggesting that exogenous xenosurveillance may be useful in estimating apparent prevalence of host–pathogens.

There are caveats to the methods we used to estimate prevalence which can be biased in multiple ways. Germane to this study, prevalence estimates could be biased because of inaccurate estimates of the sampled host population size. This parameter could be biased by mosquito behavior in two ways. In the present study, we assumed that one blood meal represented one host individual, but sampled host population size could be underestimated if a mosquito fed multiple times from different hosts and thus multiple individuals were represented in a single blood meal. Alternatively, the sample population size of hosts could be overestimated in the blood meal samples if multiple mosquitoes fed on the same animal. For example, a turkey hen incubating eggs or a neonatal deer may remain relatively motionless for extended periods of time and, thus, be more likely to be fed upon by large numbers of mosquitoes. Such individual hosts may be overrepresented in a sample if mosquitoes were collected near a site where a host had been sessile. We attempted to mitigate these potential biases by creating sampling events that were spatially and temporally independent of one another, but the actual sampled population size was unknown in our study. To truly validate the ability of exogenous xenosurveillance to accurately estimate apparent prevalence of host–pathogens, molecular methods such as SNP or microsatellite assays that discriminate individual vertebrate hosts could be deployed simultaneously with pathogen xenosurveillance to determine the number of hosts that were represented in the blood meal samples. With this approach, if hosts were represented more than once in a sampling event, the sampled population size could be adjusted and the replicated samples used to assess pathogen detectability to more accurately estimate prevalence [36]. Future studies should evaluate the utility of SNP or microsatellite assays in quantifying sampled host population size.

Although the majority of mosquitoes collected were not blood-fed, approximately one in 12 mosquitoes was engorged. Our study shows that with sufficient mosquito collection effort, a large sample of vertebrate peripheral blood can be collected, far more than if an equivalent effort of animal capture techniques were used to directly sample any of the vertebrates targeted in this study. In many cases exogenous xenosurveillance may complement existing efforts to sample vertebrates that are otherwise difficult to capture and handle, either because of low density (e.g., endangered species), their cryptic nature (e.g., invasive reptiles such as chameleons), or when vertebrates reside in remote and difficult terrain (e.g., forest canopies).

Xenosurveillance is a broad term used to incorporate two distinct types of surveillance: the first definition seeks to detect and survey known or potential pathogens in arthropod vector species transmitted either mechanically or biologically. For example, several studies have aimed to determine if African swine fever virus can be transmitted by hematophagous insects, including mosquitoes [37,38]. These studies did not target blood engorged mosquitoes; the methods they used targeted insects that were host-seeking including one study that purposefully avoided blood-fed mosquitoes because researchers were trying to determine if biological transmission could occur [38]. The present study had a decidedly different goal: to use blood-fed mosquitoes to determine if vertebrate hosts circulated pathogens in their peripheral blood stream, and none of these pathogens were mosquito-borne. To avoid confusion about the goals of xenosurveillance studies, we suggest that future studies acknowledge these differences by referring to them as endogenous xenosurveillance which focuses on the role that the sampled vector has in transmission versus exogenous xenosurveillance which focuses on the epidemiology of vertebrate pathogens based on an arthropod blood meal and does not invoke the sampled arthropod species as their vectors.

## Data Availability

All data are contained within the text of the manuscript or in Supplementary files.

## Supplementary Materials

The following supporting information can be downloaded at: https://www.mdpi.com/article/10.3390/pathogens14080792/s1. Supplementary File provides methodological details on the amplification of viral nucleic acid. Supplementary Table S1 details the number of blood meals collected for each host species during this study, the mosquito species containing these blood meals and the totals for the dry and wet season. Supplementary Table S2 details the results of the pathogen screening of the samples from each host blood meal, season and mosquito species. Supplementary Table S3 provides the Ct values for blood meals that were positive for BTV nucleic acid.

## Abbreviations

The following abbreviations are used in this manuscript:

DNA	Deoxyribonucleic acid
TTSuV1	Torque teno sus virus 1
LPDV	Lymphoproliferative virus
BTV	Bluetongue virus
RNA	Ribonucleic acid
USA	United States of America
CO_2_	Carbon dioxide
FTA	Flinders Technology Associates
PBS	Phosphate-buffered saline
PCR	Polymerase chain reaction
TAE	Tris-Acetate-EDTA
qPCR	Quantitative polymerase chain reaction
RT-qPCR	Real time quantitative polymerase chain reaction
SNP	Single nucleotide polymorphism
