# Supplementary material for: Evaluation of Mosquito Blood Meals as a Tool for Wildlife Pathogen Surveillance
Source: Pathogens. 2025 Aug 8;14(8):792. doi: 10.3390/pathogens14080792 (PMC12389132; doi:10.3390/pathogens14080792)
Supplement: Supplementary file 1 [file pathogens-14-00792-s001.zip › Supplementary file.pdf]

Supplementary file

Method details for amplification of viral nucleic acid

Torque teno Sus Virus 1 - TTSuV

For each 20 ul PCR reaction, we included 10uL of 2x Qiagen Taq PCR MasterMix (Qiagen Puregene, Qiagen, Hilden, Germany), 1uL of forward primer (10uM stock concentration) (0.5uM reaction concentration) (Table 1), 1uL of reverse primer (10uM stock concentration) (0.5uM reaction concentration) (Table 1), 0.4uL of $MgCl_2$ (25mM stock solution) (2.0mM reaction concentration) (Promega Biotechnology Company, Madison, Wisconsin, USA), 3.6uL of molecular grade water, and 4uL of the extracted DNA template. PCR amplification was performed using an Eppendorf Mastercycler ProS (Eppendorf, Hamburg, Germany). The amplification conditions were as follows, an initial denaturation step of 94C for 5 minutes, followed by 40 cycles of 94C for 15 seconds, 54C for 20 seconds, and 72C for 30 seconds, with a final elongation step of 72C for 5 minutes.

Lymphoproliferative Virus - LPDV

For each reaction a 25uL mixture was created using 12.5uL of 2x Qiagen Taq PCR Master Mix (Qiagen Puregene, Qiagen, Hilden, Germany), 0.5uL of forward primer (10uM stock concentration, 0.2uM reaction concentration, Table 1), 0.5uL of reverse primer (10uM stock concentration, 0.2uM reaction concentration, Table 1), 1uL of MgCl2 (25mM stock solution, 2.5mM reaction concentration, Promega Biotechnology Company, Madison, Wisconsin, USA), 8.5uL of molecular grade water, and finally 5uL of the extracted DNA template. The amplification conditions were as follows, an initial denaturation step of 94C for 3 minutes, followed by 35 cycles of 94C for 30 seconds, 54C for 30 seconds, and 72C for 60 seconds, with a final elongation step of 72C for 5 minutes.

Bluetongue Virus - BTV

For each sample, a reaction mixture of 25uL was created using 12.5 2x AgPath One Step RTPCR buffer, 1uL of AgPath 25x OneStep RTPCR Enzyme (Thermo Fisher Scientific, Waltham, Massachusetts, USA), 0.5uL of forward primer (10uM stock concentration, 0.4uM reaction concentration, Table 1), 0.5uL of reverse primer (10uM stock concentration, 0.4uM reaction concentration, Table 1), 0.1uL of fluorescently labeled probe (FAM, Table 1). In addition, 0.1uL of VetMAX Xeno Internal Positive Control RNA (Thermo Fisher Scientific, Waltham, Massachusetts, USA) and 0.8uL of VetMAX Xeno Internal Positive Control – VIC (Thermo Fisher Scientific, Waltham, Massachusetts, USA) were added as an internal amplification positive control to identify internal PCR inhibition. Finally, 5.5uL of water was added to the master mix and 4uL of template RNA was added to each reaction. All amplifications were performed on a QuantStudio 5 quantitative PCR machine (Thermo Fisher Scientific, Waltham, Massachusetts, USA).

Table 1: Primer sequence for molecular detection of TTSuV1, LPDV, and BTV from mosquito blood meals with associated citation.

Pathogen:	Citation:	Primer Sequence:
TTSuV1	Segales et al., 2009<br>Martinez et al., 2009	TTV1F 5' – CGGGTTCAGGAGGCTCAAT – 3'<br>TTV1R 5' – GCCATTCGGAACTGCACTTACT – 3'
LPDV	Allison et al., 2014<br>Alger et al., 2015	LPDVF 5′ – ATGAGGACTTGTTAGATTGGTTAC – 3′<br>LPDVR 5′ – TGATGGCGTCAGGGCTATTTG – 3′
BTV	Wernicke et al., 2015	BTV-NS3-183F 5' – AAATMTTGGAYAAAGCRATGTCAAA – 3'<br>BTV-NS3-288R 5' – CTYACRTCATCACGAAACGCT – 3'<br>BTV-NS3-242FAM 5' – FAM-AAR GCT GCA TTC GCA TCG TAC GC-BHQ1 – 3'
